# Prior Knowledge Predicts Early Consolidation in Second Language Learning

**DOI:** 10.3389/fpsyg.2019.02312

**Published:** 2019-10-14

**Authors:** Dafna Ben Zion, Michael Nevat, Anat Prior, Tali Bitan

**Affiliations:** ^1^Department of Learning Disabilities, University of Haifa, Haifa, Israel; ^2^Edmond J. Safra Brain Research Center for the Study of Learning Disabilities, University of Haifa, Haifa, Israel; ^3^The Language and Brain Plasticity Lab, Institute of Information Processing and Decision Making, University of Haifa, Haifa, Israel; ^4^Department of Psychology, University of Haifa, Haifa, Israel; ^5^Department of Speech Language Pathology, University of Toronto, Toronto, ON, Canada

**Keywords:** learning, consolidation, morphology, individual differences, second language

## Abstract

Language learning occurs in distinct phases. Whereas some improvement is evident during training, offline memory consolidation processes that take place after the end of training play an important role in learning of linguistic information. The timing of offline consolidation is thought to depend on the type of task, with generalization of implicit knowledge suggested to take more time and sleep to consolidate. The current study aims to investigate individual differences in the timing of consolidation following learning of morphological inflections in a novel language in typical adults. Participants learned to make plural inflections in an artificial language, where inflection was based on morpho-phonological regularities. Participants were trained in the evening, and consolidation was measured after two intervals: 12 h (one night) and 36 h (two nights) post training. We measured both inflection of trained items, which may rely on item-specific learning, and generalization to new untrained items, which requires extraction of morpho-phonological regularities. The results for both trained and un-trained items showed two patterns of consolidation: early versus late, that is while some participants improved during the first night, others, who deteriorated in performance during the first night, improved in the later consolidation interval. Importantly, phonological awareness in L1 predicted *early* consolidation for trained items. Furthermore, there was no association between participants’ consolidation trajectory in trained and untrained items. Our results suggest that consolidation timing depends on the interaction between task characteristics and individual abilities. Moreover, the results show that prior meta-linguistic knowledge predicts the quality of early consolidation processes. These results are consistent with studies in rodents and humans, showing that prior knowledge accelerates consolidation of newly learnt episodic memory. Finally, the rate of consolidation across exposures to the language might explain some of the variability found in the attained level of second language proficiency.

## Introduction

Learning a second language is a difficult and prolonged endeavor, with large variability in learning rate, trajectory and gained proficiency (Roberts, 2012; Dornyei, 2014). Language learning also occurs in distinct phases. Whereas some improvement occurs on-line, during exposure and training, there is also evidence for consolidation processes, which manifest as off-line gains after the end of training (Rasch and Born, 2013; James et al., 2017). Most previous research has focused on individual differences in online language learning, and less is known about possible individual differences in consolidation of novel linguistic information. The current study addresses this issue in the context of an artificial language learning task, of novel morphological inflections, previously shown to rely on fronto-striatal mechanisms (Nevat et al., 2017). Specifically, we examined individual differences in consolidation of trained items and untrained items which involves the ability to extract regularities from the input, and their possible links to individual differences in meta-linguistic abilities.

Memory consolidation is the process by which a new, initially fragile memory is transformed into a stable memory after the end of training (Robertson et al., 2004). This process is time dependent and embodied in synaptic and cellular modifications of brain circuits (Dudai et al., 2015). Behaviorally it is indicated by offline improvement in performance or stabilization of learning gains, in the absence of additional training (Stickgold et al., 2001; Fenn et al., 2003). Consolidation can be apparent following a single consolidation period, but some knowledge requires longer periods of 48 h to 4–7 days and even weeks to complete offline improvement (Berchtold et al., 2010; Dayan and Cohen, 2011; Sawangjit et al., 2018). The neurocognitive mechanism underlying consolidation may be task dependent. According to the “active systems consolidation” concept, episodic memories which involve hippocampal encoding, consolidate during sleep through replay in the hippocampus that results in enhancement of neocortical associations (Diekelmann and Born, 2010). This is the process by which the newly learned information is integrated into overarching networks, and new schemata are formed (Vicari et al., 2003; Lewis and Durrant, 2011; Rasch and Born, 2013; Stickgold and Walker, 2013). In contrast, skill learning relies on activation in neocortical regions during the initial acquisition phase. While there is a debate regarding the degree to which consolidation of neocortical learning is sleep dependent (Klinzing et al., 2019; Lerner and Gluck, 2019), recent evidence suggests that consolidation of some neocortical skills may also involve hippocampal replay during sleep (King et al., 2017; Sawangjit et al., 2018; Lerner and Gluck, 2019). According to the “information overlaps to abstract” (iOtA) model (Lewis and Durrant, 2011), consolidation induces selective strengthening of the overlapping features across separate memories, while eliminating the rest, thus forming the basis for extraction of statistical regularities and generalization of rules to new items.

Interestingly, possible individual differences in consolidation have not received much attention in the literature. Thus, typical adults and children differ in their ability to immediately recall newly learned information (Gettinger, 1984; Ackerman, 1987; Jonassen and Grabowski, 2012), but the degree to which individuals might differ in the off-line consolidation phase is much less studied. Several studies have shown weaker consolidation in atypical populations. For example, when compared to typical controls, adults with ADHD showed delayed consolidation and less off-line gains in a motor task (Adi-Japha et al., 2011) and children with ADHD also showed less benefit from sleep in a picture recognition test (Wilhelm et al., 2012). Adults diagnosed with Dyslexia showed lower off line gains in motor sequence learning in some studies (Needle et al., 2015) but not others (Gabay et al., 2012). Age has also been found to affect offline consolidation. Older adults showed smaller offline gains and no sleep dependent consolidation compared to younger adults in a motor sequence learning task (Wilson et al., 2012). Finally, individuals’ general cognitive abilities were found to affect the consolidation process. Thus, adults with high working memory capacity (WMC) showed greater off-line gains in recall of word pairs than did those with low WMC (Fenn and Hambrick, 2012). Another study reported that general intelligence was related to gains following sleep in a paired-associates memory paradigm (Fenn and Hambrick, 2015). These studies demonstrate individual differences in consolidation of declarative memory after one night of sleep, which are linked to differences in cognitive abilities. The current study focuses on individual differences in consolidation of a linguistic task which was previously found to rely on fronto-striatal mechanisms (Nevat et al., 2017).

Offline consolidation plays an important role in learning of linguistic information as well. Davis and Gaskell (2009) adapted the Complementary Learning Systems (CLS) framework (McClelland et al., 1995; McClelland, 2013) to word learning, suggesting that a newly learnt word is initially stored as a distinct episodic trace in the hippocampus, but following an offline consolidation period it becomes integrated with existing vocabulary in neocortical long-term memory. Several studies have shown the benefit of an off-line consolidation period for the integration of newly learnt words into the existing lexicon (Dumay and Gaskell, 2007; Tamminen et al., 2010). The effect of off-line consolidation is also found in grammar learning in infants exposed to an artificial language (Gomez et al., 2006; Hupbach et al., 2009) and in adults learning new morphological suffixes of real words, suggesting that generalization to untrained words required an offline consolidation period of at least one night (Davis and Gaskell, 2009; Tamminen et al., 2012; Leminen et al., 2016). In a recent study, the effect of morphological regularity on the consolidation of novel inflections was tested (Mirković et al., 2019). Mirković et al. (2019), reported an increase in the tendency to apply the irregular suffix following a consolidation period of 12 h independent of sleep, with further increase after 24 h (Mirković et al., 2019). Interestingly, the authors found no increase in application of a default morphological inflection (a suffix with high type frequency and no phonological cues), concluding that such learning may be more dependent on neocortical mechanisms. Similar to studies of non-linguistic learning, these studies have mostly tested gains following a single consolidation interval for each participant.

Language learning and specifically second language learning, varies widely between learners (Robinson, 2003; Benati and VanPatten, 2015). Thus, learners’ cognitive and linguistic abilities serve as powerful predictors for second language learning success (e.g., Ganschow et al., 1998; Koda, 2007); for reviews on this extensive research field see Zafar and Meenakshi (2012), Dornyei (2014), Fillmore et al. (2014). Understanding the factors contributing to individual variability in language learning is important for two main reasons. Theoretically, identifying the contribution of L1 knowledge and skills to L2 learning can inform and ground theories of transfer and the neural and cognitive overlap between first and additional languages (Abutalebi, 2008; Chung et al., 2018). Practically, understanding how existing skills of learners might impact their individual learning trajectory can lead to improved teaching methods, tailored to specific learner profiles. One ability identified as making significant contributions to success in second language learning is phonological awareness, the awareness and the ability to manipulate the smallest sound units in a word (Melby-Lervåg and Lervåg, 2011). For example, phonological awareness in children’s L1 predicts their word learning in L2 (Hu, 2014), and adults with a phonological processing deficit in L1 showed low second language performance in L2 (Borodkin and Faust, 2014). Second language learning can also be predicted by other linguistic abilities, for instance L1 morphological awareness. Thus, children’s ability to decompose real words in their L1 contributes to their vocabulary knowledge and word reading in L2 (Ramirez et al., 2010; Pasquarella et al., 2011).

In line with this extensive body of research supporting individual differences in online language learning, several studies have also documented individual differences in offline gains when learning linguistic materials. For example, L1 phonological processing abilities were found to predict sleep dependent consolidation of discriminating an unfamiliar phonological contrast (Earle and Arthur, 2017). Along similar lines, learners with larger vocabularies in their first language showed greater consolidation in integrating newly learned words into their existing lexicon (James et al., 2017). Finally, participants’ L1 word reading ability was positively correlated with their offline improvement when learning to read in an artificial orthography (Bitan and Booth, 2012). These findings suggest that participants’ prior linguistic knowledge may affect their consolidation process when learning a second language.

Artificial language paradigms, like the one used in the current study, are particularly well suited for examining learning and generalization because one can tightly control the amount and type of language exposure, by manipulating factors of interest in the input. Artificial linguistic paradigms have the added advantage that they can generally be learned to reasonably high proficiency over the course of hours to days. Hence, despite concerns regarding their ecological validity because they do not reflect the full complexity of natural languages, artificial languages have been widely used to investigate learning of both vocabulary (Davis and Gaskell, 2009; Tamminen et al., 2012) and grammar (Ellis and Schmidt, 1997; Merkx et al., 2011; Morgan-Short et al., 2012a, b). Importantly, performance on artificial language learning has been found to correlate positively with natural second language learning (Ettlinger et al., 2016), and training on an artificial language can result in native-like brain activity patterns (Morgan-Short et al., 2012a, b). In the present study, learning of the artificial language was used as a model of second language learning.

Previous research has found individual differences in many aspects of language learning, including artificial languages, mostly in online measures of learning. In the current study, we ask whether such individual differences can also be found in the time course of consolidation of new linguistic information. Further, we examine whether such individual differences in consolidation might be linked to differences in the meta-linguistic skills of phonological and morphological awareness. Hence, we examine consolidation over two intervals following learning of morphological inflections which include both item-specific information and learning of regularities.

We used an artificial language learning paradigm (Bitan et al., 2013; Nevat et al., 2017, 2018) in which participants learned five pseudo plural suffixes that were applied to singular pseudo stems based on the stems’ final syllable (phonological cue) (see Table 1). The paradigm also introduced variability in suffix frequency, so that participants learned suffixes that appeared with high, medium or low frequency. Following the exposure phase, participants were tested both on their ability to correctly assign a suffix to the stems they encountered during exposure, and on their ability to assign suffixes to novel stems which included the same phonological cues (extraction of regularities). Participants were not explicitly informed of the morpho-phonological regularities governing the suffix selection based on the phonological cue. However, our previous studies showed that participants were able to extract the rule from the input, particularly for the high frequency suffix (Nevat et al., 2017, 2018). Each participant completed one training session in the evening, and two testing sessions, occurring in the mornings 12 and 36 h post training. This schedule that included two consolidation intervals (first night, second day+night) allows us to track possible individual differences in the extended time line of consolidation.

**TABLE 1:** List of trained items.

	**High-frequency suffix**			**Medium-frequency suffix**			**Low-frequency suffixes**		
	“-an”			“-esh”			“-ev,” “-ak,” “-ur”		
	(Applied to 18 items)			(Applied to nine items)			(Applied to nine items: three suffixes × three items)		

**Deterministic cues**	**Deterministic cues**			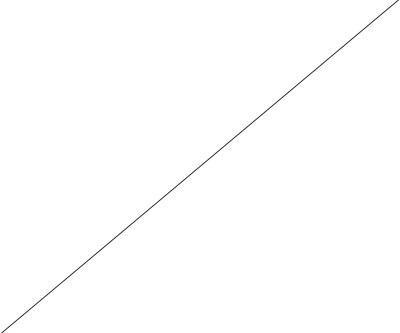			**Deterministic cues (unique)**		
	Three groups of four items each:						Unique suffixes:		
	tuvoz	givig	bikul				“-ev”	“-ak”	“-ur”
	nifoz	bolig	mupul				deipem	nerud	rinit
	gishoz	rekig	tedjul				sapor	lidek	getav
	laloz	posig	tizul						

**Non-Deterministic cues**	**Non- deterministic cues**			**Non- deterministic cues**			**Non- deterministic cues**		

	Three items with cues consistent with the medium frequency suffix:			Three groups of three items each:					
	Shalod (-an)	gukiv (-an)	gitun (-an)	moshod	sibiv	batun	Three items with cues shared with items taking the high frequency suffix:		
	Three items with cues consistent with a low frequency suffix:			resod	paniv	ligun	Meshus (-ev)	Shibil (-ak)	Zufom (-ur)
	kunus (-an)	gomil (-an)	pakom (-an)	napod	tepiv	rosun			

*Presented by suffix frequency (high, medium, and low).*

Our research questions were: (1) Are there individual differences in the timeline of consolidation when learning morphological inflections, especially for the high frequency suffix? (2) Do learners show the same individual pattern of consolidation in the inflection of trained and untrained items, which differentially rely on item specific learning and extraction of regularities? (3) Is the individual pattern of consolidation predicted by the learners’ cognitive abilities and prior knowledge?

## Materials and Methods

### Participants

Twenty healthy young adults, 24–34 years old (mean = 27.85, SD = 2.63, 10 women) participated in the study. All participants were native Hebrew speakers and spoke at least one other language (English) as a foreign language, with no history of neurological or psychiatric illness, learning disability or attention disorder, no addiction to alcohol, and were non-smokers. All had normal or corrected hearing and vision. All reported a regular sleep schedule, with habitual sleep duration between 6 and 9 h. Exclusionary criteria to ensure good quality of sleep include: use of medication that affects sleep; taking mid-day naps; pregnancy; working night shifts; *trans*-Atlantic trips 3 months prior to the study; excessive caffeinated beverages drinking per day; obesity (BMI > 30, group mean = 22.49). Sleep disorders were ruled out by a questionnaire based on the Epworth Sleepiness Scale (ESS) for daytime sleepiness and the Berlin Questionnaire for Sleep Apnea (Johns, 1991; Netzer et al., 1999). All participants were “morning types” or “moderately morning types,” as assessed by the Hebrew version of the Morningness-Eveningness Questionnaire (MEQ) (Horne and Östberg, 1976), MEQ scores 59 to 72, mean = 64.27). Participants maintained at least 6 h of proper nocturnal sleep during the three nights prior to the experiment, as reported in a sleep log. During the last 24 h and during the experiment itself (that lasted 36 h) they abstained from caffeinated and alcoholic drinks.

Participants reading level was ascertained by two screening tests: One-minute Word reading test (mean 122.83; 14.52 SD), and Pseudoword reading test (mean 66.22; 7.67 SD) (Shatil, 1995, 1997). In these tests participants read a list of pointed words or pseudowords as quickly and as accurately as possible within 1 min and the number of correct items was counted. Participants were excluded if they scored less than one standard deviation below the average of our local norms (Weiss et al., 2015) in both tests. To test the effect of individual differences on consolidation, additional baseline parameters were measured. Since the regularity within the artificial language was based on morpho-phonological cues, phonological and morphological awareness parameters were measured. Participants completed a Phoneme Deletion Test for Pseudowords (mean accuracy, 92.2%; 5.56% SD, mean total time 76.22; 7.67 SD) (Ben-Dror and Shani, 1996), in which they listened to 25 pseudowords and were instructed to omit a specified phoneme located at the beginning or middle of a given pseudoword. The time to respond in this test together with the score on the 1 min pseudoword reading test were used to calculate a composite phonological awareness score for each participant. This was done by converting both scores to standardized scores (*Z* score) and averaging between them (*M* = 0.176; SD = 0.88). In addition, morphological awareness was tested with a word-inflection task (mean accuracy, 85.46%; 6.79% SD, mean total time 76.22; 7.67 SD), which included 26 items, in which participants were asked to produce a bound morphological form out of two words (Cohen-Mimran, 2009).

### Materials

The task and stimuli were adapted from Nevat et al. (2017, 2018). Trained items consisted of 36 novel words, which were aurally presented together with pictures of objects they refer to. All items consisted of two syllables (CVCVC) in their singular form (the stem). Plural forms were created by applying one of five possible (VC) suffixes to the stem. The suffixes differed in frequency so that the high-frequency suffix was applied to half of the items (18 items), the medium frequency suffix was applied to one quarter of the items (9), and three low frequency suffixes were applied to one twelfth (3) of the items. Our predictions and the main analyses in the current study focus on words taking the high frequency suffix, that have shown most of the effects in previous studies (Bitan et al., 2013; Nevat et al., 2018). However, for the completeness of the report we start from a group analysis of suffixes from all frequencies.

Pairings of stems and suffixes were mostly determined by the last two phonemes of the stem, the phonological cue. For example, stems ending with/oz/took the high-frequency suffix (“–an”); thus, the plural for “tuv**oz”** was “tuvoz**an.”** For the low frequency suffixes, none of the stems had an identical cue, making each word receiving these suffixes unique. However, some trained items did not follow these rules, reflecting the inconsistency of natural languages. These “exception” words, which contained “non-deterministic cues,” took a different suffix from the one that was predicted by the word’s cue. For example, although most words ending with the cue “od” received the suffix “-esh,” the stem “shalod” received the suffix “-an” forming the word “shalodan” which did not adhere to the general inflection rule. Thus, of the 18 words receiving the high frequency suffix 12 words contained deterministic cues (-oz, -ig, -ul, which were always associated with the suffix “-an”), and six words contained non-deterministic cues (e.g., –od, which was associated with both “-an” and “-esh”). All words receiving the medium frequency suffix contained non-deterministic cues. For words receiving the low frequency suffixes, six contained deterministic cues (these cues were unique to a single word receiving a low frequency suffix) and three words contained non-deterministic cues. Participants were not informed of any of the patterns underlying stem-suffix pairings (see Table 1).

Participants were also tested on the inflection of untrained items. A list of 96 additional items, not presented during training, was presented in each of the three transfer tests. Forty-eight of the 96 words in each transfer test had a deterministic cue consistent with the trained items, 24 had non-deterministic cues, and 24 words did not have any cue contained in the trained items. Only items containing deterministic cues were analyzed in the current study.

### Procedure

**FIGURE 1:**
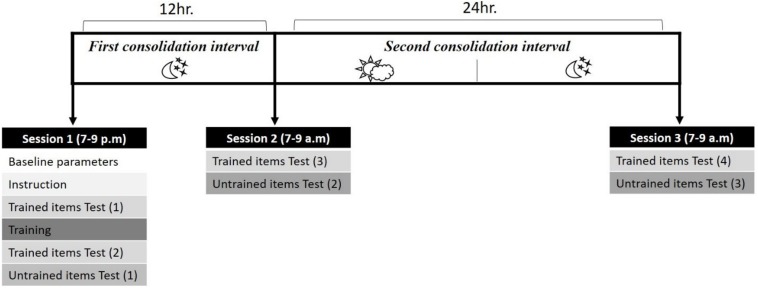
Overall design of the experiment.

The experiment took place over the course of three sessions (over 36 h in total) in a setting of evening-morning-next morning: (1) A training session during which baseline parameters were taken and the artificial language was taught and tested; this session took place at the sleep laboratory during the evening (7–9 p.m). Following the first session, participants slept at the sleep laboratory for polysomnographic parameter recording (data not reported here). (2) The second session was held in the morning (at 7 a.m), at the sleep laboratory. (3) The third and final session was held the next morning (36 h post training, 7–9 a.m) in participants’ homes (see Figure 1).

#### Instruction Block

**FIGURE 2:**
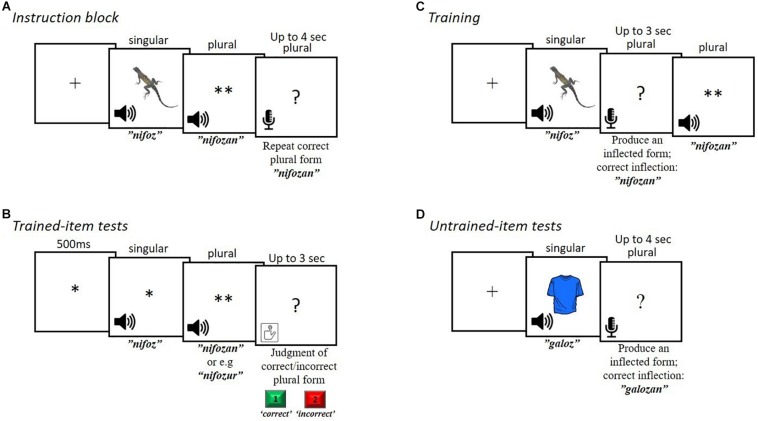
Design of trials. **(A)** Instruction block: each item was presented once, together with the picture that refers to their meanings. **(B)** Trained-item tests: each item was tested once and required the judgment of correctly and incorrectly inflected plural forms. **(C)** Training: participants produced the inflected forms of the trained items receiving feedback. **(D)** Untrained-item tests (transfer-test): participants inflected untrained items from their singular to their plural form.

The first session began with an instruction block in which each of the 36 training items was presented once. When the participant pressed a key, the singular form was presented aurally together with an image of a real object it referred to (e.g., an apple) on the screen. The singular form was followed by a visual cue consisting of two asterisks (^∗∗^), indicating the plural form of the word would soon be presented. The plural form was then presented aurally, followed by the presentation of a question mark, indicating that participants were to repeat the plural form they had just heard. The question mark remained on the screen for a maximal duration of 4 s, or until a vocal response was detected (see Figure 2A).

#### Trained Item Tests

Trained-item tests requiring the judgment of correctly and incorrectly inflected plural forms were presented both before and after training in the first session, and in each of the following two sessions (four trained-item tests overall). Each of the 36 trained items was tested once in each test. The singular form was presented (without its picture) followed by an aurally presented plural form of the same word. Participants were instructed to press “1” on a standard keyboard if the plural form was correct, and press “2” if not. They were given 3 s to respond (see Figure 2B). In each test, half of the 36 plural forms presented were correct, and half were incorrect. Incorrect inflections were created by adding one of the other suffixes to the stem. The order of the presentation was counterbalanced across participants. Across sessions, each participant was presented with all different incorrectly affixed forms.

#### Training

Training took place in the first session (see Figure 1), following the instruction block. During training, participant heard the singular form accompanied with its picture and attempted to produce the plural form. The correct plural form of the word was then presented aurally, as feedback (Figure 2C). The training session consisted of four blocks, in which each trained item was presented once. The order of items within each training block was randomized.

#### Un-Trained Items Tests

In these tests participants were asked to inflect 96 untrained items from their singular to their plural form (Figure 2D). Words were presented in a randomized order. The untrained item test was presented at the end of each session, for a total of three times. For each transfer test the 96 untrained items were different.

### Statistical Analysis

#### Trained Items

Response times (RT) for correct responses were calculated. Two participants whose mean RT was beyond the range of ±2.5 SD were excluded from the analysis, for a final sample of 18 participants. Percent of correct responses was calculated for each individual in each frequency at each testing point. The normality of the distribution of these measures across individuals was tested for skewness and kurtosis. Separate two-way repeated measures ANOVA were conducted on accuracy and RT as dependent measures, with frequency (including low, medium, and high frequency) and four testing time points (pre- training, end of training, 12 h, and 36 h post training) as within subject factors. Since our previous studies (Bitan et al., 2013; Nevat et al., 2017, 2018) revealed strong effects of learning and consolidation for trained and untrained items inflected with the high-frequency suffix subsequent analyses of the consolidation intervals focused on these items.

#### Un-Trained Items

To test whether participants became sensitive to the phonological cues embedded in the trained items and their ability to generalize this sensitivity to untrained items, we calculated for each participant their “sensitivity to phonological cues” by calculating the participant’s accuracy in inflecting untrained items which contained deterministic cues, minus the participant’s tendency to apply that suffix to untrained items with no cue. This measure ranges from a maximum sensitivity of 100 (perfect accuracy and no application of the suffix to words with no cues) to a minimum of −100 (zero accuracy and the suffix was applied to all words without cues). The sensitivity score was computed separately for high and low frequency suffixes (there were no deterministic cues for the medium frequency), at each testing time.

## Results

### Trained Items – Judgment Task

**FIGURE 3:**
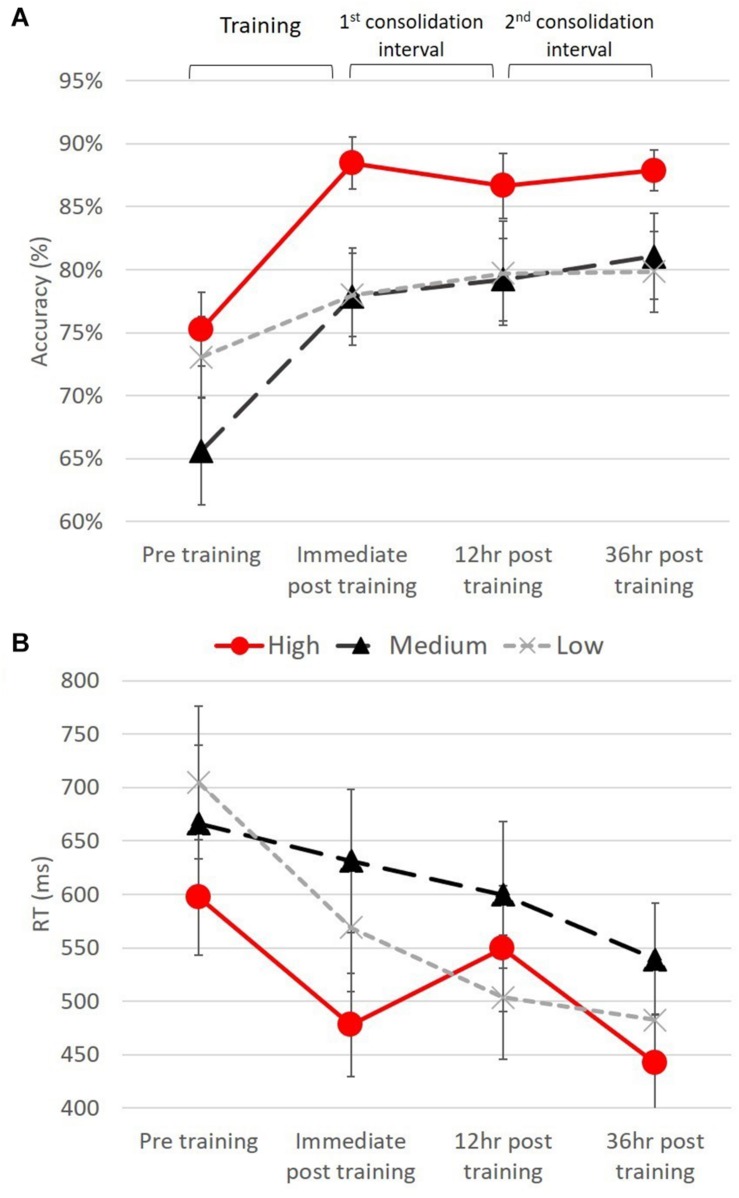
Learning curves for trained items, by frequency. Performance, **(A)** accuracy and **(B)** reaction time are presented on the four trained-item tests.

The distributions of the accuracy measures for each frequency level and test were within the acceptable range of skewness (min. −0.943; max. 0.339) and kurtosis (min. −1.082; max. 0.337), fulfilling the normality assumptions for ANOVA. A two-way repeated measures ANOVA was used to analyze participants’ accuracy rates (in percent) with suffix frequency (high, medium, low) and testing time point (pre-training, immediately after training, 12 h post training, and 36 h post training) as the within-subject variables (see Figure 3A). The main effect of test was significant [*F*(3,51) = 7.23, *p* < 0.001], as participants’ accuracy across all frequencies improved from pre training test to post training test and was maintained after 12 h and after 36 h. Pairwise comparisons between tests showed that performance on the pre-training test was lower than in all other tests (all *p* < 0.01), which did not differ from each other. The analysis also showed a significant main effect of suffix frequency [*F*(2,34) = 9.00, *p* < 0.005], with higher accuracy on items receiving the high-frequency suffix than on items receiving either medium- or low-frequency suffixes (both *p* < 0.005), which did not differ from each other.

An analogous two-way repeated measures ANOVA on average RTs showed significant main effects of test, [*F*(3,51) = 5.54, *p* < 0.005] and suffix frequency [*F*(2,34) = 5.21, *p* < 0.05]. Pairwise comparisons between suffix frequency levels showed that performance on items receiving the high frequency suffix was significantly faster than for items receiving the medium frequency suffix (*p* < 0.01) and marginally faster than for items receiving low frequency suffixes (*p* = 0.051) (see Figure 2C). Given our specific prior hypotheses regarding learning of the high frequency inflection we conducted a separate one-way ANOVA within each suffix frequency, with test as a within subject factor.

These analyses revealed a significant main effect of test for the high [*F*(3,51) = 5.00, *p* < 0.005]; and low [*F*(3,51) = 6.99, *p* < 0.001] frequency suffixes, but not for the medium frequency suffix [*F*(3,51) = 1.39, *p* = 0.255]. For the low frequency suffix, performance improved during training, and was maintained throughout the following tests, with significant pairwise differences between pre-training test and all other tests (all *p* < 0.05). In contrast, for the high frequency suffix, RT did not show a smooth pattern of maintenance. Pairwise comparisons showed a significant difference between pre-training test and immediate post-training test (*p* < 0.05) reflecting improvement in RT during training and between pre-training test and 36 h post training test (*p* < 0.01), indicating that improvement was maintained after 36 h. Interestingly, RTs in 12 h post training test did not differ from RTs in pre-training test (*p* = 0.36) but were significantly slower than RTs in the immediate post training test (*p* < 0.05) and 36 h post training test (*p* < 0.05). Hence, performance on the trained items receiving the high frequency suffix improved during training, became slower during the first 12-h consolidation interval, and improved back during the second consolidation interval (see Figure 3B).

**FIGURE 4:**
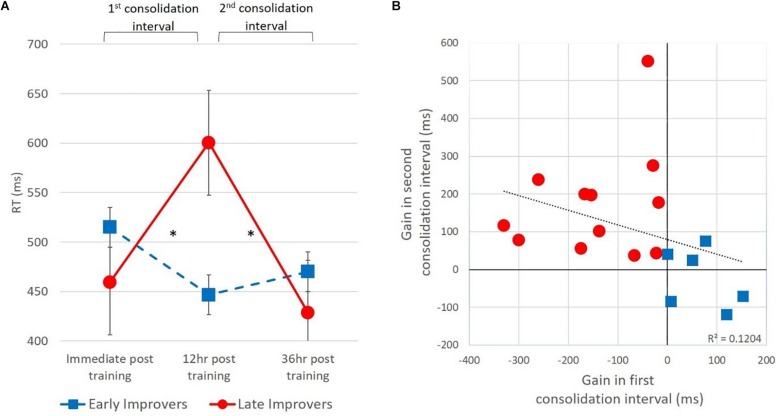
Offline gains in RT for trained items inflected with the high frequency suffix. **(A)** RT in the three post-training tests, presented for subgroups divided based on above or below zero gain in the first consolidation interval. **(B)** correlation across groups between RT gains during the first and second consolidation intervals.

In order to understand whether individual differences in timing of consolidation can explain this unexpected pattern in the high frequency suffix, we computed for each participant their gain in RT for each consolidation interval. Thus, the first consolidation interval was computed as the difference in RT between immediate post-training test and the 12 h post training test. The second consolidation interval was computed as the difference in RT between 12 h post training test and the 36 h post training test. The computation of these consolidation gains revealed that a subset of the participants (*N* = 6) improved during the first consolidation interval and maintained their performance during the second interval, and were therefore termed “early improvers”, whereas the remaining participants deteriorated in performance speed during the first consolidation interval and improved during the second interval, termed “late improvers” (*N* = 12) (see Figure 4A).

To examine whether the two groups we identified indeed differed from each other statistically, we conducted a two-way ANOVA on RTs with group and Test (immediate post training and 12 h post training) as the independent variables, and found a significant interaction of group by test [*F*(1,16) = 18.31, *p* < 0.005]. Follow up comparisons showed significant improvement during the first consolidation interval for “early improvers” [*t*(5) = 2.74, *p* < 0.05] and significant deterioration during this interval for late improvers [*t*(4) = −4.41 *p* < 0.005]. To examine performance in the second consolidation interval, we conducted a two-sample *T* test to compare between groups and found a significant effect of group [*t*(16) = −3.72, *p* < 0.005], such that the “late improvers” showed greater improvement than the “early improvers” in the second interval. The improvement of the “late improvers” in the second interval was significant [*t*(11) = 4.162, *p* < 0.005], and they reached the same level of performance in the final test as the “early improvers” [*t*(16) = 0.52, *p* = 0.610], who maintained their performance during the second interval. Finally, the Pearson correlation between the first and second consolidation interval scores computed across all participants was negative, but did not reach statistical significance [*r*(18) = −0.347, *p* = 0.158] (see Figure 4B).

**FIGURE 5:**
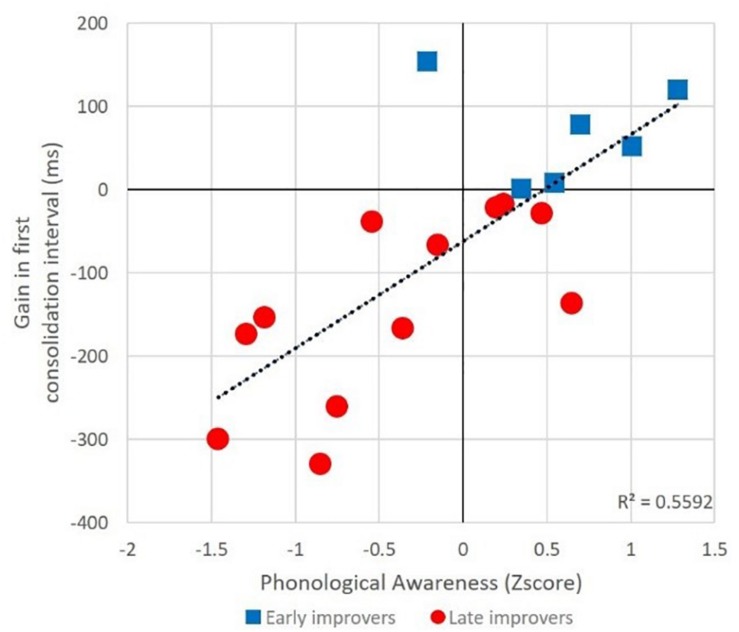
Correlation between phonological awareness and RT gains during the first consolidation interval in trained items inflected with the high frequency suffix.

Finally, to test the hypothesis that linguistic abilities might explain consolidation gains, we tested the correlation between participants’ consolidation gains in the first and second interval and their phonological and morphological awareness scores. We found a strong positive correlation, between the phonological awareness composite score and improvement during the first consolidation interval [*r*(18) = 0.745, *p* < 0.001] (see Figure 5). No correlation was found for the morphological awareness measures, nor for the second consolidation interval.

### Un-Trained Items – Transfer Task

**FIGURE 6:**
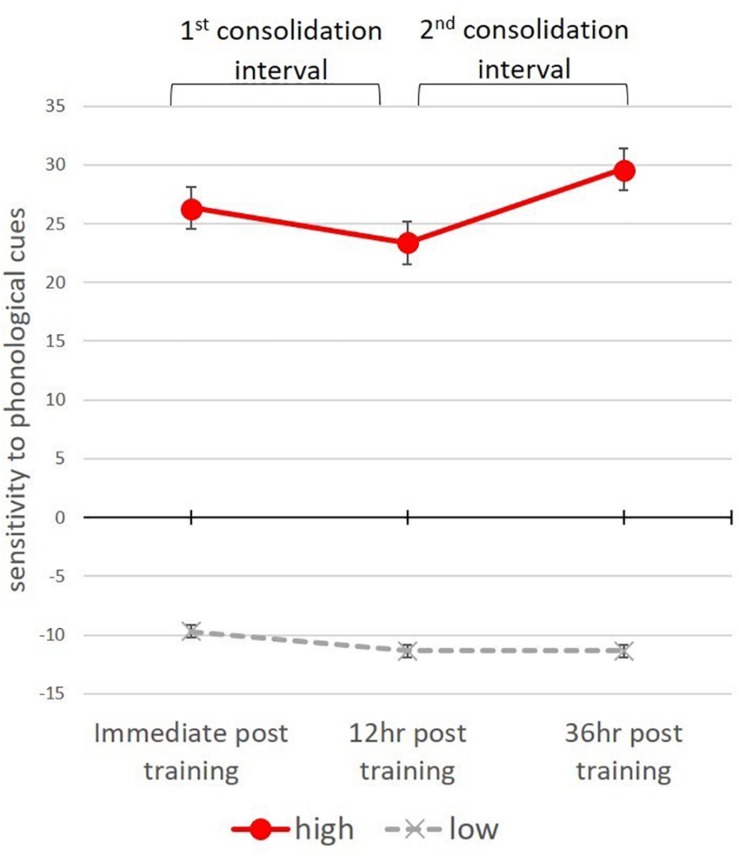
Sensitivity to phonological cues in untrained items, inflected with the High and low frequency suffixes.

Participants’ “sensitivity to phonological cues” was calculated by subtracting each participant’s tendency to select a given suffix for untrained items with no phonological cues, from their tendency to select this suffix in the context of a deterministic cue. This was calculated for the high and low frequency suffixes at each of the three testing time points (i.e., immediately after training, 12 h. and 36 h post training). To examine whether participants were overall sensitive to the regularity of phonological cues we calculated for each participant the mean sensitivity across the three tests separately for items with high and low suffix frequencies. One sample *T*-tests showed that participants’ sensitivity to the phonological cue was significantly greater than zero in items inflected with the high frequency suffix [*t*(17) = 8.54, *p* < 0.001], and significantly lower than zero in items inflected with the low frequency suffixes [*t*(17) = −5.63, *p* < 0.001]. This indicates that participants tended to apply the low frequency suffixes more often to words containing no phonological cues, than to words that contained cues. The sensitivity to phonological cues was compared in a two-way repeated measures ANOVA with suffix frequency (low vs. high frequency) and three testing time points as within subject factors. The analysis showed a significant main effect of frequency [*F*(1,17) = 95.11, *p* < 0.001], with higher sensitivity to phonological cues in items receiving the high-frequency suffix than in items receiving the low-frequency suffixes, with no difference between tests [*F*(2,34) = 0.887, *p* = 0.42] (see Figure 6).

**FIGURE 7:**
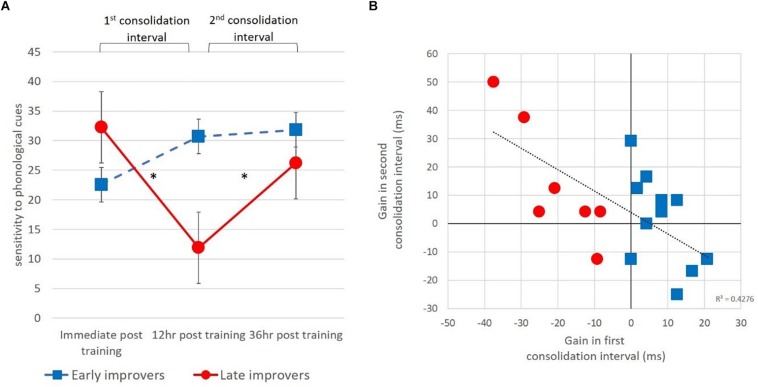
Offline gains in sensitivity to phonological cues for un-trained items inflected with the high frequency suffix. **(A)** Presented for subgroups divided based on above or below zero gain in the first consolidation interval. **(B)** A negative significant correlation between the gains in the first and second consolidation intervals, across both sub-groups.

We next examined the pattern of consolidation for untrained words with the high frequency suffix with the same analyses performed on trained items. For each participant we computed the gain in sensitivity to phonological cues obtained during the *first consolidation interval* (from immediately after training to 12 h post training), as well as the gain in sensitivity during the *second consolidation interval* (from 12 h post training to, 36 h post training). Again, we divided the participants into two groups based on their numerical gain in sensitivity to phonological cues in the first consolidation interval. Eleven participants showed numerical improvement in sensitivity to phonological cues in the first interval, whereas seven participants showed numerical deterioration in sensitivity to phonological cues during the first interval (see Figure 7A).

To test whether the gain in sensitivity to phonological cues during the two consolidation intervals are related to each other we compared the gain in the second interval between the two groups defined based on the first interval. Although there was no significant difference between the groups [*t*(16) = −1.37, *p* = 0.19], Pearson correlation analysis between the gains in the first and second consolidation intervals, across both groups was negative and significant [*r*(18) = −0.654, *p* < 0.005] (see Figure 7B). This negative correlation suggests that the consolidation process occurs in different time intervals for different individuals. Therefore, participants who improve in the early interval, do not improve in the later interval, and vice versa. In contrast to our findings for the trained items, we did not find an association between the gains during each of the consolidation intervals with either phonological or morphological awareness (all *ps*’ > 0.05).

Finally, we examined whether participants showed the same timing of consolidation across both tasks. To this end, we conducted a Pearson Chi-Square test, and found no association between participants’ pattern of consolidation in the two tasks [χ^2^(1, *N* = 18) = 0.46, *p* = 0.49].

## Discussion

The current study investigated individual differences in the consolidation process of novel linguistic information, across 36 h, and explored whether such differences might relate to prior knowledge. We found that all participants improved in accuracy and speed of inflecting trained words during training, with the best performance obtained for words inflected with the high frequency suffix. However, whereas accuracy for applying the high frequency suffix was maintained for 12 and 36 h post training, the gains in reaction time, were not maintained in the first 12-h consolidation interval, and only recovered 36 h post training. Further analysis revealed that this pattern varied considerably across individuals. Some participants improved during the first consolidation interval, and maintained their performance thereafter, whereas other participants deteriorated during the first consolidation interval and improved only during the second consolidation interval, reaching the same level of proficiency at the final test. We further found that participants with high phonological awareness showed greater offline improvement during the first consolidation interval, but no association was found between prior linguistic knowledge and improvement during the second consolidation interval.

Participants’ sensitivity to regularities in phonological cues was measured by their performance on inflection of untrained items. Participants developed this sensitivity only for words inflected with the high frequency suffix. Here too, we observed two patterns of consolidation, participants who improved during the first consolidation interval maintained their performance throughout the following interval, but individuals who showed a reduction in sensitivity during the first consolidation interval recovered during the second interval. A negative correlation was found between the gains in the first and second intervals. Importantly, there was no association between participants’ consolidation trajectory in trained and untrained items.

### Individual Differences in Consolidation Timing

Our results, showing offline gains in inflection of trained and untrained words 12 h (one night) and 36 h (two nights) after training, are broadly consistent with previous studies showing offline consolidation gains on novel language learning tasks (Davis and Gaskell, 2009; Tamminen and Gaskell, 2013; James et al., 2017). Previous studies also showed that offline improvement on many of these linguistic tasks was sleep dependent (Dumay and Gaskell, 2007; Tamminen et al., 2010). Our previous study (Bitan et al., 2013), that used the same stimuli as the current study, also showed sleep dependent consolidation. The current study did not examine the effect of sleep on consolidation, however, because training for all participants was conducted in the evening, and performance was examined in the mornings 12 and 36 h post training. Thus, the current design provided the conditions for sleep dependent consolidation during both of our consolidation intervals. Moreover, whereas most previous studies examined participants 12 and 24 h post training (Dumay and Gaskell, 2007; Tamminen et al., 2012), or several days after training (Tamminen et al., 2010, 2012, 2015), the design of the current study enabled us to identify individual differences in the *timing* of consolidation, with some individuals showing offline gains already 12 h (one night) after training, while others showed them only after 36 h (which included two nights of sleep).

Several previous studies investigated variability in consolidation processes between typical and atypical populations. For example, adults and children diagnosed with ADHD and adults diagnosed with Dyslexia who were trained on motor tasks showed lower offline gains compared to controls (Adi-Japha et al., 2011; Wilhelm et al., 2012; Needle et al., 2015). Older age was also shown to affect the strength of offline gains and consolidation with some tasks (Wilson et al., 2012). Moreover, in a study that examined learning of artificial vocabulary in adolescents and young adults, older age and good reading skills were found to modulate consolidation after one-night sleep and this was reflected in brain activation in the precuneus/posterior cingulate (Landi et al., 2018). The current findings add to these results by showing the variability within the typical young adult population, and by showing variability not only in the amount of offline gains but also in the timing. By measuring performance after two consecutive nights, we were able to demonstrate that participants who did not benefit from the first consolidation interval, did so during the second interval.

Our results, showing individual differences in the timing of consolidation while learning a novel language, are also consistent with a wide body of research showing individual differences in second language learning, as measured by the level of proficiency attained at the end of training (Dornyei and Skehan, 2003; Sanz, 2005; Dornyei, 2006, 2014). These differences are typically explained by differences in proficiency in the first language (Melby-Lervåg and Lervåg, 2011), differences in background demographic factors, such as age and gender (Long, 1990; Young and Oxford, 1997), and differences in cognitive and linguistic parameters, such as aptitude, motivation, cognitive style and learning strategies (Zafar and Meenakshi, 2012; Dornyei, 2014; Fillmore et al., 2014). Here, by addressing a less studied aspect of second language learning, i.e., offline consolidation, we show that the timing of consolidation can also vary considerably among healthy adult individuals. This variability in consolidation, after a single training session in a novel language, may explain some of the individual variability in the level of proficiency attained after long-term practice in a second language. Previous studies have shown that consolidation involves extracting the commonalities across distinct experiences (Rasch and Born, 2008, 2013; Lewis and Durrant, 2011; Stickgold and Walker, 2013). The timing of consolidation may affect the accumulation of experiences across practice sessions (Smith and Scarf, 2017), and thus affect long term performance in a second language.

### Timing of Consolidation Across Different Tasks

Our results for both trained and un-trained words revealed variability in the timing of consolidation. In both tasks there was a group of early improvers, who improved during the first consolidation interval, and maintained their performance through the next 24 h, and another group of late improvers, who deteriorated during the first consolidation interval and improved during the second one. Nevertheless, there was no association between individuals’ patterns of consolidation across tasks, suggesting that they might rely on different learning processes. Whereas performance on trained items may rely on both item-specific learning and on extracting the predictive regularities between phonological cues and suffixes, correct inflection of un-trained items is more specifically associated with extraction of morpho-phonological regularities implicitly embedded in the trained words. Note that another difference between the measures of trained and un-trained items is the task. Whereas for the trained items we used a receptive judgment task, for the generalization we used a production task.

Previous studies of linguistic learning suggest that while item-specific knowledge is evident immediately after training (Tamminen et al., 2015), generalization to un-trained items, which reflects implicit rule-extraction, required more time or sleep for consolidation (Fenn et al., 2003; Tamminen et al., 2012; Batterink et al., 2014). Theoretical accounts suggest that explicitly acquired knowledge may be rapidly learned by hippocampal and medial temporal mechanisms, and be expressed immediately after training (Born and Wilhelm, 2012). In contrast, implicit integration of newly learned knowledge with existing knowledge may depend on slower neocortical learning that requires time for consolidation (Davis and Gaskell, 2009). Moreover, a recent study (Mirković et al., 2019) found a dissociation between regular and irregular morphological inflections, with greater effects of offline consolidation on strengthening the irregular inflection. It should be noted, however, that unlike the “regular” inflection in Mirković et al. (2019) study, that had high type-frequency and was not associated with specific phonological cues, the high-frequency suffix in the current study is associated with highly predictable phonological cues.

In contrast to the above studies, our results did not show a dissociation between performance on trained and untrained items at the group level, although these tasks differentially rely on item specific vs. implicit extraction of regularities. Hence, in both tasks we found both early and late improvers. Nevertheless, it is important to note that the generalization tasks used in some of these previous studies required mapping of new information into existing information. For example, generalization was determined by the degree to which novel pseudo words interfered with the retrieval of similar real words (Davis and Gaskell, 2009) or by the application of trained morphemes to un-trained real words (Tamminen et al., 2012, 2015). In contrast, the current generalization task requires the application of morpho-phonological regularities to un-trained pseudowords, and not to real words in participants’ L1 (Hebrew).

Our findings therefore suggest that the timing of consolidation does not depend solely on the type of task, and that both item-specific learning and implicit extraction of regularities can show faster or slower consolidation processes in different individuals. However, the finding that there was no association between the consolidation patterns across the two tasks also suggests that the speed and efficiency of the consolidation process is not a consistent characteristic of the individual either. Therefore, consolidation timing does not depend exclusively on either the task characteristics or on the individual abilities but may be a result of the specific interaction between them.

### Prior Knowledge as a Predictor of Consolidation Timing

Our results for the trained-items show that participants’ offline gains during the first consolidation interval were positively correlated with their phonological awareness in L1. Participants with good phonological awareness improved more (or deteriorated less) during the first 12 h interval after training, while no correlation was found for the second consolidation interval. Previous studies in second language learning have shown that participants’ phonological awareness in their native language was a good predictor of their reading comprehension (Melby-Lervåg and Lervåg, 2011) word naming (Hu, 2014) and general proficiency (Borodkin and Faust, 2014) in their second language. In the current study, good phonological awareness may play a role in the ability to create new phonological representations of the trained words and suffixes. Alternatively, phonological awareness can reflect more general meta-linguistic abilities, which may contribute to learning of vocabulary and grammar in a novel language.

Regardless of the specific causal role of phonological awareness in our task, these findings suggest that good linguistic abilities are related to efficient early consolidation in a linguistic task. These findings are consistent with a small number of language learning studies that showed the effect of prior knowledge on consolidation. Thus, phonological abilities in L1 predicted identification and discrimination of a non-native phonetic contrast following a 12 h period of sleep after training (Earle and Arthur, 2017). L1 vocabulary predicted the offline integration of newly learned words into the existing lexicon overnight (James et al., 2017), and L1 word reading predicted the offline gains in reading a novel artificial orthography (Bitan and Booth, 2012). Furthermore, a recent study demonstrated that L1 reading skill and vocabulary knowledge were associated with improved novel word consolidation, and with the size of the consolidation effect on activation in the posterior cingulate/precuneus (Landi et al., 2018).

The current findings, showing the association between prior linguistic knowledge (phonological awareness) and efficient early consolidation of novel words and their inflections, are in line with recent developments in the consolidation theory of hippocampus related episodic memory (van Kesteren et al., 2012; Fernández and Morris, 2018). Studies in rodents show that newly learnt information that is consistent with existing schema is consolidated more rapidly and becomes independent of the hippocampus earlier than schema un-related information (Tse et al., 2007, 2011). Human studies show an increase in connectivity between the hippocampus and neocortical areas following encoding of new information related to prior knowledge (Liu et al., 2018). This effect of schema-relatedness on consolidation was associated with increased spindle density during sleep and faster decrease in hippocampal activity following encoding (Hennies et al., 2016). Other studies suggest a special role for medial prefrontal areas in the consolidation of schema related learning (van Kesteren et al., 2012).

In analogy to episodic memory studies, in the current study, individuals with good phonological, or other metalinguistic abilities in L1, may have had enhanced connectivity in specific cortical language areas, enabling the newly acquired representations of trained words and suffixes to be more readily incorporated into these neocortical networks. Alternatively, high prior linguistic abilities may enable participants to quickly consolidate the more predictable associations between cues and suffix and disregard all other associations. However, more assumptions would be needed to explain why such prior phonological ability did not correlate with the consolidation of untrained items. It should be noted that our previous fMRI study that examined learning of the same artificial language (Nevat et al., 2017) did not find hippocampal involvement in the inflection of trained words, measured immediately after a single training session, but showed activation in lateral and medial frontal cortices and in the caudate nuclei, which decreased with more training (Nevat et al., 2017). Nevertheless, given that trained words are initially learnt as specific items, we cannot exclude the possibility that this task also involves the hippocampus in very early stages of encoding (Breitenstein et al., 2005) or conversely, during the consolidation phase. Indeed, recent studies suggest that even learning of skills that are not hippocampal dependent during initial phases of acquisition may show hippocampal based sleep dependent consolidation (King et al., 2017; Sawangjit et al., 2018; Klinzing et al., 2019). The findings of the current study extend previous findings on the consolidation of episodic memory to show that prior knowledge also affects consolidation of new *linguistic* information that presumably relies on fronto-striatal learning mechanisms. This idea also receives support from recent findings of an artificial language learning study showing that individual differences in reading ability predicted the effect of consolidation on activation in the posterior cingulate gyrus/precuneus (Landi et al., 2018).

The current study should be viewed in light of several limitations. First, the number of participants in the study was rather small (*N* = 18). Thus, further research should aim to replicate these findings. Second, during the first consolidation interval participants slept in a sleep lab (polysomnographic measures not reported here). Although this could have affected the quality of their sleep, sleep efficiency assessed by the sleep laboratory, was found to be satisfactory (*M* = 88.8, SD = 6.22) and no correlation was found between sleep efficiency and offline gains in any of the tasks. The third limitation of the current study is the absence of correlations found with the morphological awareness test. Although our training was focused on morphological inflections, only the phonological awareness composite score was associated with consolidation. Nevertheless, our findings cannot lead to a conclusive interpretation regarding the role of morphological awareness in learning novel morphological inflections because morphological awareness is a multifaceted variable (e.g., Tighe and Schatschneider, 2015; Goodwin et al., 2017). In the current exploratory investigation, we included only a single measure of morphological awareness, tapping only one aspect of this meta-linguistic ability. Thus, this issue is ripe for a fuller investigation in future studies, which could include a wider array of morphological awareness and processing measures (e.g., Apel et al., 2013; Shahar-Yames et al., 2018). Along similar lines, because we only examined phonological and morphological awareness, we cannot determine at this stage whether phonological awareness in itself is an ability which influenced the consolidation, or alternatively, whether higher metalinguistic abilities of different kinds may also support efficient consolidation. Thus, another direction for future investigations is to tap into the possible contribution of metalinguistic abilities more generally (e.g., syntactic awareness) to individual differences in consolidation of novel linguistic information.

## Conclusion

By examining the consolidation of novel words and their morphological inflections in a healthy adult population on two consecutive days, the current study identified individual variability in the timing of consolidation, with early and late offline improvers. This variability was identified both in a task that relies on implicit extraction of statistical regularities, and in a task that also involves item-specific learning. These tasks were shown in a previous study to rely on cortical and fronto-striatal learning mechanisms (Nevat et al., 2017). The current results show that the timing of consolidation is determined by the specific interaction between the properties of the language learning task and the characteristics of each individual. One property that affects the individual’s timing of consolidation is their prior knowledge relevant to the task at hand. Thus, our results show that an individual’s L1 phonological awareness (in itself or as a proxy for general meta-linguistic abilities) may facilitate early consolidation of the second language. Previous studies suggest that the timing of consolidation may determine the effectiveness of subsequent training sessions, through the process of re-consolidation (Smith and Scarf, 2017), and thus affect the accumulation of linguistic knowledge in long term learning. Thus, the association between phonological awareness in L1 and proficiency in L2 may be mediated by the effect of prior knowledge on the timing of consolidation. While these findings are in line with recent developments in hippocampal dependent episodic memory (Tse et al., 2011; McClelland, 2013; Fernández and Morris, 2018) our findings extend this to a language learning task that relies on fronto-striatal mechanisms.

## Data Availability Statement

The raw data supporting the conclusion of this manuscript will be made available by the authors, without undue reservation to any qualified researcher.

## Ethics Statement

This study involving human participants was reviewed and approved by the Ethics Committee of the University of Haifa and by the Helsinki ethics committee of the Rambam Health Care Campus. All participants provided their written informed consent to participate in this study.

## Author Contributions

TB and MN conceived the study. DB collected the data. DB, AP, and TB analyzed the data and wrote the manuscript.

## Conflict of Interest

The authors declare that the research was conducted in the absence of any commercial or financial relationships that could be construed as a potential conflict of interest.
